# Species pool distributions along functional trade-offs shape plant productivity–diversity relationships

**DOI:** 10.1038/s41598-017-15334-4

**Published:** 2017-11-13

**Authors:** Loïc Chalmandrier, Camille Albouy, Loïc Pellissier

**Affiliations:** 1 Landscape Ecology, Institute of Terrestrial Ecosystems, ETH Zürich, Zürich, Switzerland; 2 Swiss Federal Research Institute WSL, 8903 Birmensdorf, Switzerland; 3 IFREMER, unit “Ecologie et Modèles pour l’Halieutique”, rue de l’Ile d’Yeu, BP21105, 44311 Nantes cedex 3, France

## Abstract

Grasslands deliver the resources for food production and are among the most biologically diverse ecosystems. These characteristics are often in conflict as increasing yield through fertilization can lead to biodiversity loss. Thus, the challenge in grassland management is to sustain both yield and diversity. Biodiversity–ecosystem functioning experiments typically reveal a positive relationship between manipulated species diversity and productivity. In contrast, observations of the effect of increasing productivity via fertilization suggest a negative association with biodiversity. Using a mathematical model simulating species co-existence along a resource gradient, we show that trade-offs and species pool structure (size and trait distribution) determines the shape of the productivity-diversity relationship. At a constant resource level, over-yielding drives a positive relationship between biodiversity and productivity. In contrast, along a resource gradient, the shape of the productivity-diversity relationship is determined by the distribution of species along trade-off axes and often resulted in a bell-shaped relationship. In accordance to this theoretical result, we then explain the general trend of plant biodiversity loss with fertilisation in the European flora, by showing empirical evidence that trait distribution of plant species pools throughout Europe is biased toward species preferring poorer soils.

## Introduction

Grasslands provide a wealth of ecosystem services to human society, supported by species diversity and functions^1^. Biomass production (hereafter productivity^2^) in grasslands delivers the raw resources for dairy and meat production, sustaining an economy worth hundreds of millions worldwide^3^. However, current levels of exploitation might not be sustainable: to increase grassland productivity, increased use of fertilisers in grasslands leads to an overall drop of biodiversity in arable ecosystems^4,5^. Paradoxically, experimental manipulations of species in grasslands have highlighted a positive correlation between species richness and productivity^6,7^, arguing for measures to conserve biodiversity as a mean of preserving also grassland productivity. Hence, on the one hand, species diversity is expected to increase grassland productivity^8^ and this relationship seems robust to fertilization^9^. On the other hand, increased resource availability (for instance due to fertilization) results in in higher productivity and biodiversity loss^10^.

Mechanisms linking species diversity and community productivity under varying resource levels have been widely discussed^11^, but more rarely formalized within multi-species community models^12,13^. Along a resource gradient, the establishment of species from a given species pool is only possible above a minimum nutrient level^14^. Then, as resources increase further, biomass increases, but so too does competition for light, causing the exclusion of species with lower competitive abilities for light^15–17^. Species turnover along resource gradients is thus explained by trade-offs among species functions, where species able to endure low resource levels are excluded under competition for light^18^, shaping unimodal or negative relationships between diversity and productivity^19,20^. In contrast, the positive relationship between species diversity and productivity reported in biodiversity–ecosystem function (BEF) experiments^21^ is explained by niche complementarity^9,22,23^. Greater niche similarity between conspecifics leads to greater intra- than inter-specific competition, and so for a similar number of individuals, there is less competitive interference between individuals in a diverse community compared to a low-diversity community leading to a higher productivity. These alternative mechanisms provide distinct expectations for the shape of the biodiversity–productivity relationship and should be studied within a unified model.

Integrating these processes within a mechanistic mathematical model might clarify the scientific debate over a general global relationship between biodiversity and productivity that has been found to be weak^24^ despite experimental and observations showing a positive^9^, unimodal^20,25^ or negative relationships^26^. An explanation for this is that communities represent a subset of a species pool and the shape of the local relationship between diversity and productivity that arises will ultimately depend on the trait distribution within the species pool^27–29^. We hypothesize that the response of communities to resource availability should relate to the frequency of oligotrophic versus copiotrophic species in the pool (i.e. adapted to low vs. high nutrient level). This “species pool effect” has been largely overlooked by ecologists^30^ and we therefore lack theoretical predictions as to how the structure of the local species pool (number of species and the distribution of their trait values) might affect small-scale community pattern. Here, we show with a mathematical model that the link between the trait distribution within the species pool and the realized community determines the response to resource gradients, and thus unifies experimental observations (i.e. positive diversity-productivity relationships^9^) and empirical observations (i.e. variable shapes of diversity-productivity relationships^24,29^).

We present a plant–resource model that provides theoretical expectations about the causes of the shape of productivity-diversity relationships. Using this model, we show (1) that trade-offs among species traits are necessary conditions for the emergence of niche partitioning along a productivity gradient and a non-positive relationship between diversity and productivity, and (2) how the structure of the species pool affects productivity–diversity relationships. The mathematical model of plant–resource community dynamics integrates mechanisms of growth, competition, resource uptake and productivity, modulated by theoretical functional traits. The model links the quantity of a single resource, *R*, representing soil resources, to the biomass, *P*_*i*_, of plant species *i* in a pool of *N* species with the following architecture:

1 $$\frac{dR}{dt}=a(S-R)-\sum _{i}{f}_{i}{P}_{i}\,R$$

2 $$\frac{d{P}_{i}}{dt}={P}_{i}\times ({f}_{i}R-{m}_{i}-{c}_{i}{P}_{i}-{l}_{i}\sum _{j}{P}_{j})$$

The resource dynamics and plant resource absorption is akin to Tilman’s model formulation^14^; it is represented as a chemostat model, where in the absence of plants, the resource renews at a rate *a* until it reaches the maximum resource capacity *S*. The biomass of plant species *i* depends on its resource uptake ability *f*_*i*_, intrinsic mortality *m*_*i*_, intraspecific competition *c*_*i*_ and the negative effect of neighbouring biomass *l*_*i*_. Growth is modelled as a Holling type I functional response, where in the absence of interference by competitors, the relative growth rate of species *i* is proportional to the amount of resource and the species biomass. In the absence of resource and interference from competitors, the relative growth rate of species *i* is negative and its absolute value equal to the mortality rate *m*_*i*_ and the growth rate of species *i* decreases with a rate *c*_*i*_ with the biomass of its conspecifics due to greater conspecific than interspecific niche overlap (this rate was kept constant throughout the study). Competition for light was modelled in a phenomenological way: the relative growth rate of species *i* in a community decreases linearly with the total plant biomass $(\sum _{j}{P}_{j})$, at a rate *l*_*i*_ that differs among species reflecting a variable tolerance to neighbour competition for light^15,31^. The theoretical traits in the model architecture were designed to link the model to the existing knowledge about the main functional axes of plant ecological strategies^18,32^. In classical Lotka-Volterra systems, species competition is modelled through pairwise competitive interactions. While these coefficients have been linked to functional traits^33^, they do not relate easily to general knowledge that plant functional strategies are distributed along main ecological axes. Here we formulated a competition model that directly relates to Grime’s Competition-Stress axis of ecological strategy and consider established knowledge about herbaceous species competitive ability^34^.

We first explored whether functional trade-offs (no trade-off, single trade-offs and triple trade-offs) among three varying theoretical functional traits (*f*_*i*_, *m*_*i*_, *l*_*i*_) representing resource uptake, mortality, and negative effect of neighbouring biomass, could explain species turnover and the shape of the productivity-diversity relationship along resource gradients. Then, in the triple trade-off model, we manipulated species trait distributions along those trade-offs together with the size of the species pool to evaluate the consequence on the shape of the relationship between species diversity and productivity. Finally, we related our theoretical conclusions to empirical evidence that trait distributions in species pools are biased by mapping nutrient preferences for 6046 species across Europe.

## Results

### Trade-offs promote species turnover and generate bell-shaped productivity-relationship

**Figure 1:**
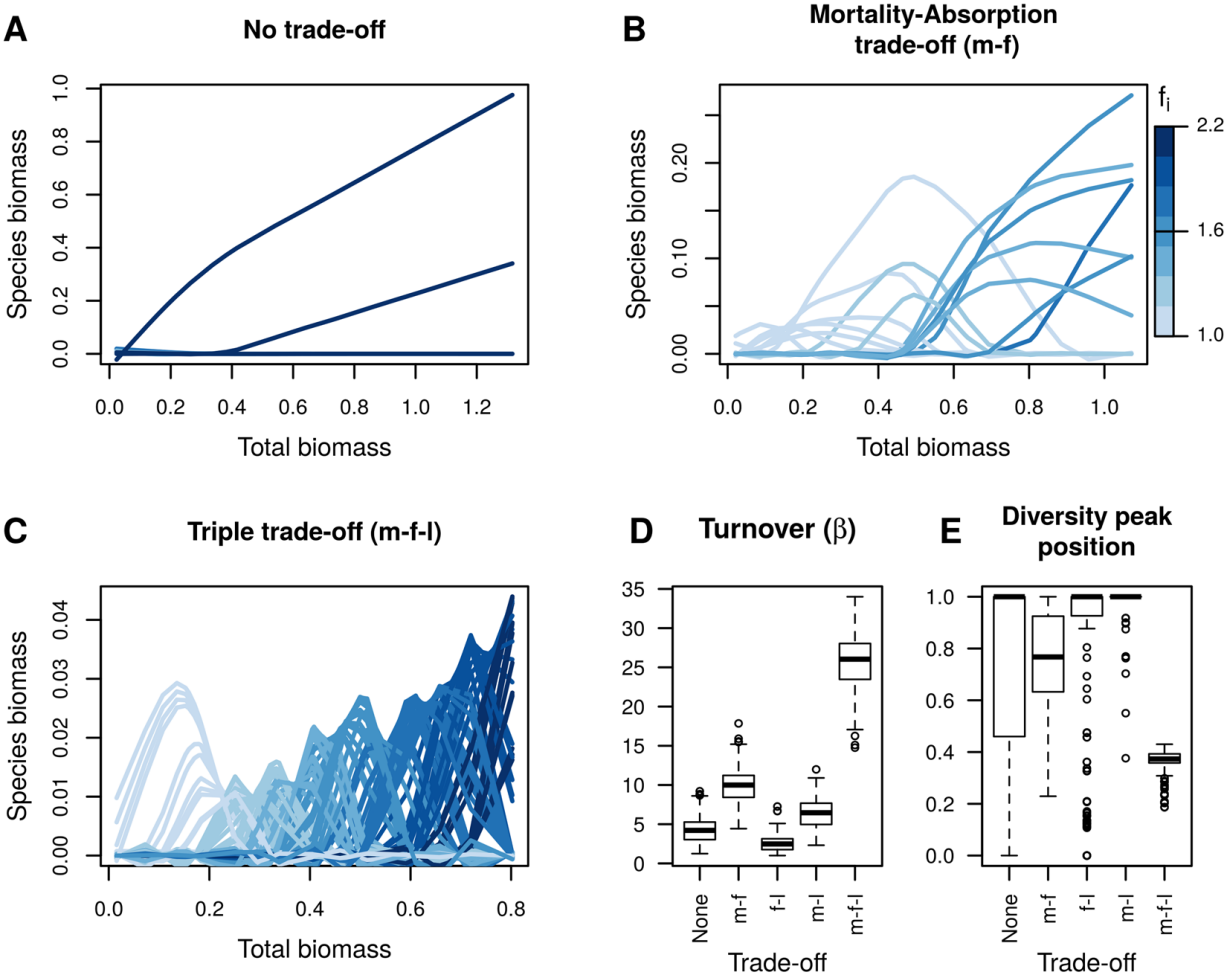
Influence of trade-offs on community turnover along a resource gradient represented by total community biomass. (**A**–**C**) Single species biomass along the gradient total community biomass; the different figures represent species pools with a different set of trade-offs among traits. The shades of blue represent the values of species f_i_ with light blue representing low values of f_i_* and dark blue representing high values of f_i_*. Other possible trade-offs (mortality–biomass tolerance, resource absorption–biomass tolerance) are displayed in the supplementary material. (**D**) turnover (β-diversity) along resource gradients as a function of the trade-offs. Parameters used to generate this figure were: N = 125, b = 0.5, average of f_i_ = 1.6, variability of f_i_ = 1.2, average of l_i_ = 2.05, variability of l_i_ = 1.7, c_i_ = 0.6, B = 0.5. Distributions of species traits along the trade-off axes were kept uniform. The assembly of 800 different species pools were simulated to generate this figure.

We simulated the assembly of communities along a resource gradient. We contrasted five different trade-offs situations: no trade-off, single trade-off (mortality-resource acquisition trade-off, mortality-sensitivity to competition trade-off or resource acquisition-sensitivity to competition trade-off) and triple trade-off (mortality-resource acquisition-sensitivity to competition trade-off). These trade-offs represent an increasing constraint of species traits along Grime’s Competition–Stress tolerance niche axis. We then compared how individual species biomass are constrained along the productivity gradient (Fig. 1A–C) and how overall community turnover (β-diversity) is affected by the number and nature of trade-offs.

We show that the inclusion of a mortality–resource absorption trade-off is necessary to generate species sorting (Fig. 1B): all species were only able to maintain positive biomass on a restricted part of the productivity gradient. This prevents the dominance of a few species performing well across the whole gradient compared to the situation where species traits were not constrained by trade-offs (Fig. 1A). This trade-off follows classical expectations of theoretical community ecology^14,18,35^ and is supported by many direct empirical evidence^32^. In contrast, a trade-off between mortality and biomass tolerance or resource uptake and biomass tolerance were not sufficient to generate species sorting along the productivity gradient (Fig. 1D).

However the mortality–resource absorption was not sufficient to explain a decrease in biodiversity at higher level of productivity (Fig. 1E). This situation only arose when sensitivity to competition was added to this trade-off, creating a bell-shaped relationship and additionally increasing species turnover along the productivity gradient. This pattern emerged because of the strict inversion of the competitive hierarchy: species more competitive in resource-poor environments are also sensitive to the high level of neighbouring biomass in resource-rich environments (Fig. 1C,D). This eventually led to their extinction in productive habitats^36^. Together, we show that multiple trade-offs (resource absorption, mortality and competitive ability) in community models explain not only species co-existence, but are also necessary to explain why the relationship between diversity and productivity is rather bell-shaped than positive.

### Species pool distribution along trade-off axes

We established that trade-offs explain community turnover along a productivity gradient. It is however expected that the distribution of species trait along trade-off axes in the species pool will also shape the response of assemblages to resource availability. Using our model of community assembly along a gradient of increasing resources, we tested how different features of the species pool can affect the shape of the productivity-diversity relationship. We focused on the number of species, the distribution of their trait values as well the average and variability of the local trait range.

**Figure 2:**
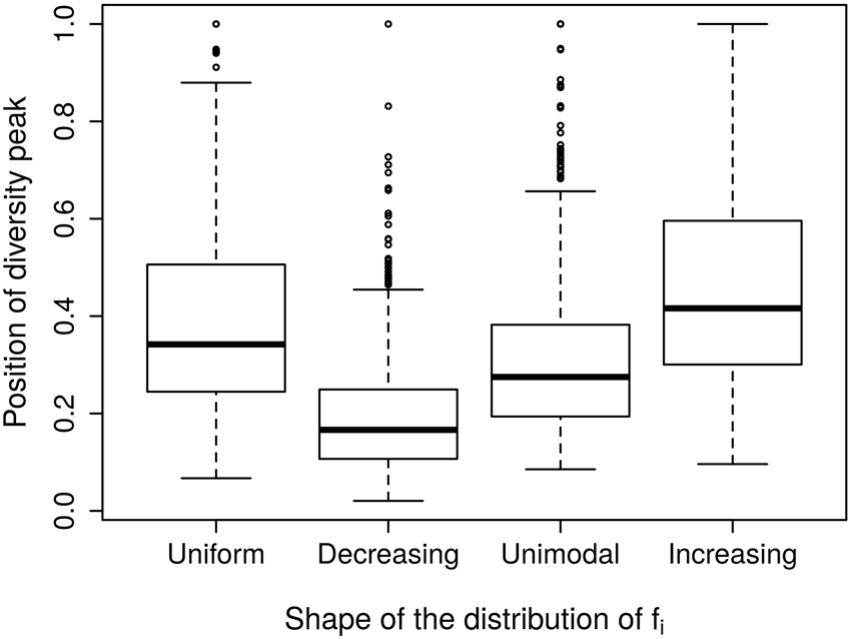
Influence of the shapes of the distribution of f_i_ values on the position of the diversity peak along the biomass gradient across all simulations. A position of 0 indicates that diversity peaks at low level of biomass while a position of 1 indicates that diversity peaks at high level of biomass.

**Figure 3:**
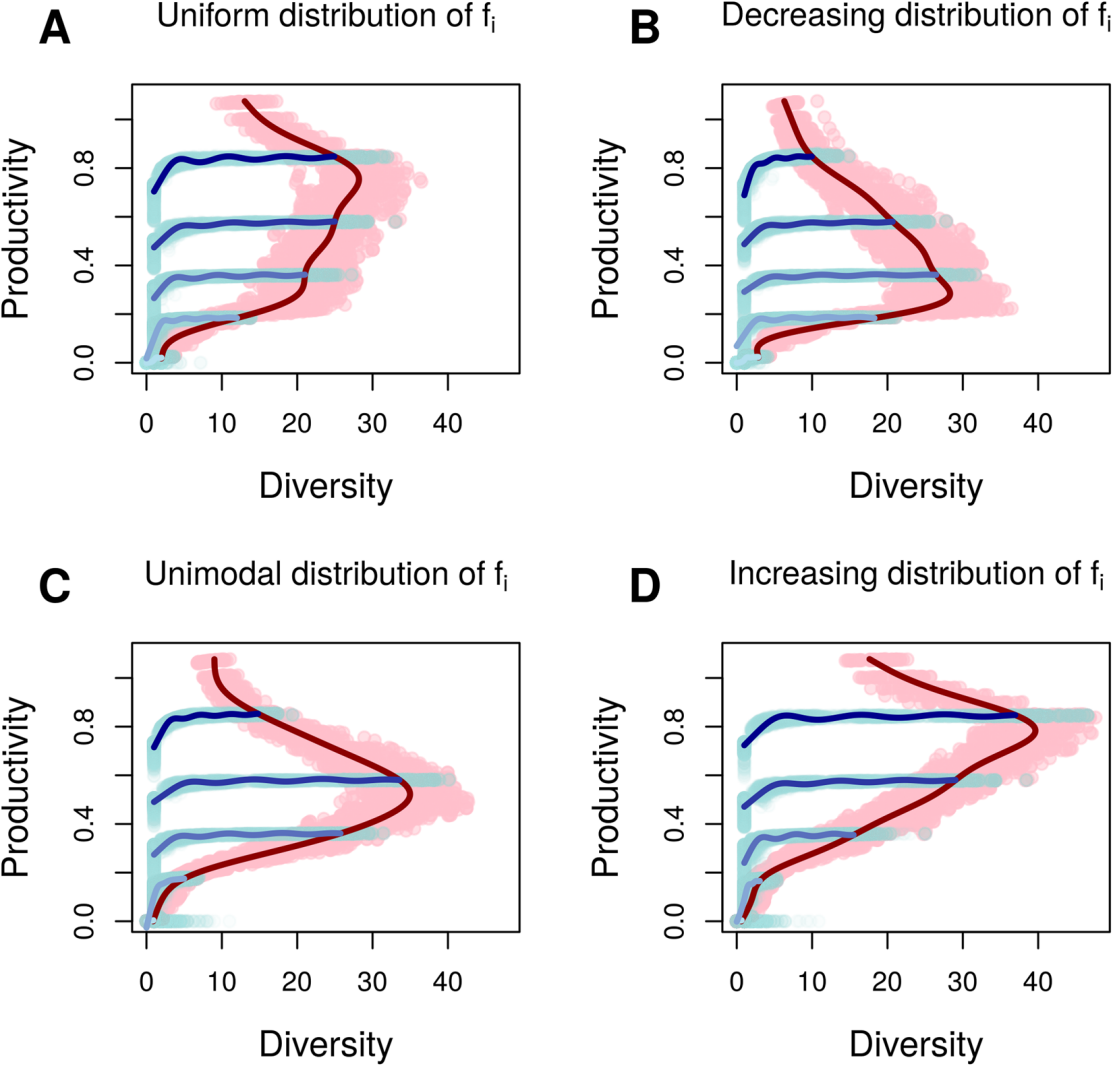
Diversity–productivity relationships for different distributions of trait f_i_. Pink points and associated modelled red curve represent the relationship along a resource gradient (increasing values of a and S). Light blue points and the associated blue curves represent simulations of BEF experiments, generated by subsampling a fixed number of species in the species pool, and this under different resource conditions (darker blue curves correspond to higher values of a and S). This figure illustrates the discrepancy between BEF experiments and observations of established communities: BEF experiments measure the increase in productivity as species diversity increases toward saturation (moving left to right along a blue line) at constant resource conditions, while observations of established or natural communities focus on gradient of soil resources (sampling a series of points on a red line). The BEF set-up produces systematic positive relationships between biodiversity and productivity, while the saturated state depends on soil resources in interaction with the structure of the species pool (the distribution of f_i_). The figures reproduce expectations of Schmid^31^. Parameters used to generate this figure were: N = 125, b = 0.5, average of f_i_ = 1.6, variability of f_i_ = 1.2, average of l_i_ = 2.05, variability of l_i_ = 1.7, c_i_ = 0.6. The assembly of 800 different species pools were simulated to generate this figure.

When combining all 3000 simulated species pool, diversity-productivity values form a cloud of values with no distinct general relationship, akin to previous empirical work^24^. However, after isolating data points generated for a given species pool structure, we generally observed bell-shaped diversity–productivity relationships with varying diversity peak position or an increasing relationship in the most extreme situations (Figs 2 and 3): species first accumulate as resource availability increases, then as the increasing amount of resources results in increased biomass, this leads to the extinction of species intolerant to biomass. This results in a decrease in diversity unless this process is compensated by the higher prevalence of copiotrophic species (high f_i_, m_i_ and low l_i_) that are then able to maintain themselves in situations of high competition.

**Figure 4:**
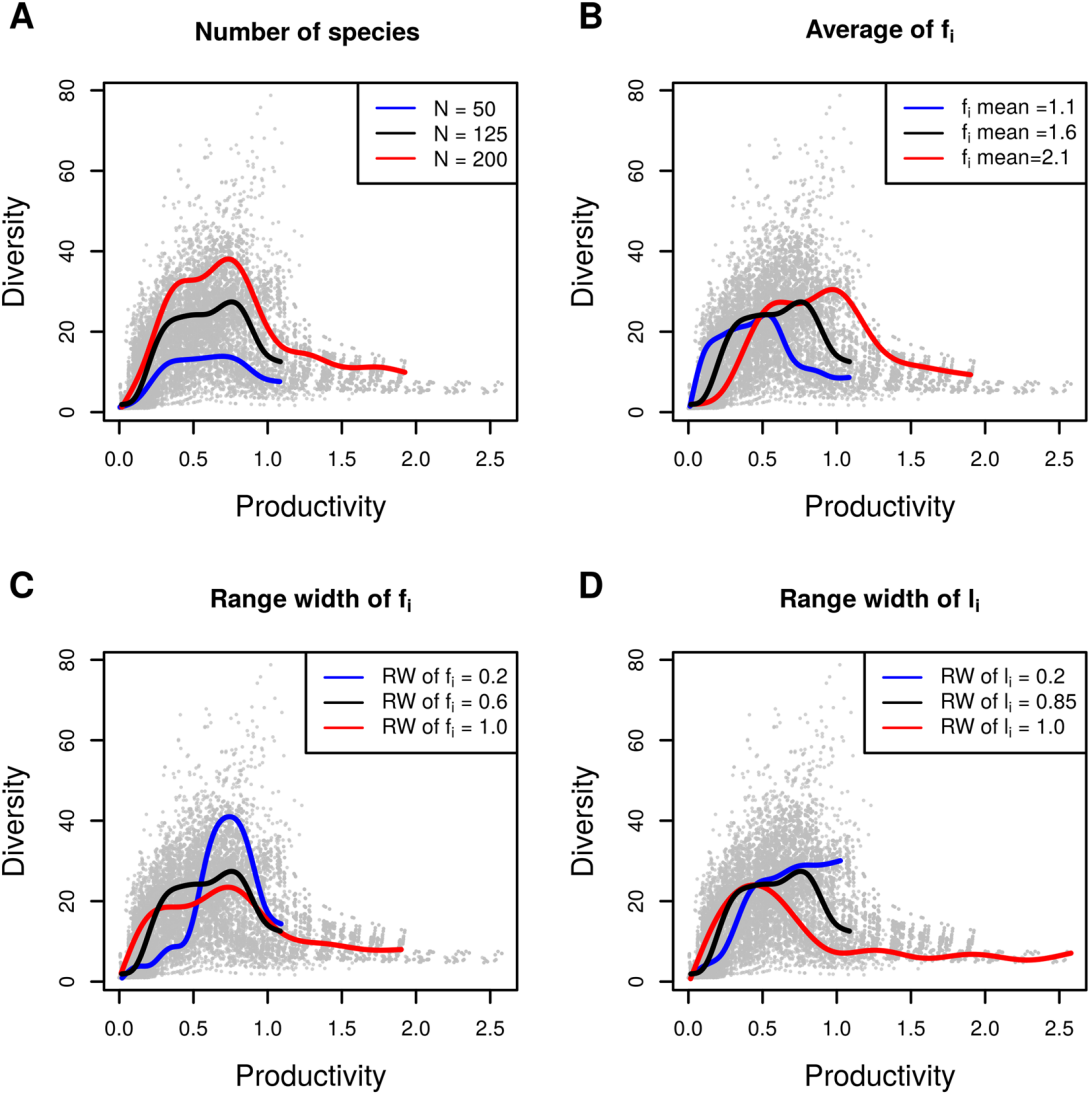
Illustration of the influence of number of species (**A**), mean of f_i_ values in the species pool (**B**), range width (RW) of f_i_ and l_i_ values in the species pool (**C** and **D**) on the shape of the diversity-productivity relationship. In each panel, the blue, black and red lines represent the diversity-productivity relationship for respectively the minimal, average and maximal values of each of these species pool features, all the other species pool being set to the average (see Table S1). The curve was estimated from 20 simulated species pools. Background points are the same across the four panels and show values of diversity and productivity along the resource gradient generated by a random subset of 500 simulated species pools across all the range of species pool feature values.

Despite these common characteristics, we found that the position of the diversity peak, the average and coefficient of variation of the diversity were affected differently by species pool structure parameters: bias in *f*_*i*_ distribution, number of species, trait variability and average of trait values (Table 1, Figs 3 and 4). Here, we only discussed only the most important factors (i,e. with a partial η^2^ superior to 0.15, Table 1):

1. Modifying the distribution of *f*_*i*_ in the species pool impacted the shape of the relationship between diversity and productivity in communities (Table 1, Fig. 2). A bias towards low values of *f*_*i*_ (e.g. a decreasing distribution of *f*_*i*_ values) and thus according to the triple trade-off, low values of *m*_*i*_, and high values of *l*_*i*_ (resp. high, high and low) generated a peak of diversity at low productivity (resp. high productivity) and a more variable diversity along the productivity gradient. A large proportion of species with high nutrient uptake ability (increasing distribution of *f*_*i*_) in the species pool led to a more variable diversity, mainly because there was a steeper increase of diversity at low levels of productivity.
2. The number of species in the species pool affected the average of the diversity along the productivity gradient (but not the position of the peak of diversity): more species in the species pool led to more species in communities (Fig. 4A).
3. The variability of traits (*f*_*i*_ and *l*_*i*_) in the species pool moderately influenced the shape of the curve. A large variability of *f*_*i*_ or *l*_*i*_ in the species pool led to a peak of diversity in less productive environments (Figs 2 and 3). Furthermore, a large variability of f_*i*_ led to less variable diversity (i.e., a flatter curve) across the productivity gradient (Fig. 4C). This can be explained by the greater contrast between strong and weak competitors fostering more competitive exclusion in moderate or high productivity environments than that seen in less variable species pools.
4. The average trait values had comparatively little influence on the diversity–productivity relationship (Table 1, Fig. 4B). When nutrient acquisition rates (f_i_) were higher on average, it led to a diversity peak at higher level of productivity and incidentally a more variable curve, because it diminished the sensitivity of all species to competitive exclusion. Species could thus co-exist until higher levels of productivity before competitive exclusion diminished diversity.

**Table 1:** Parameter estimates from a linear model linking the position of the diversity peak along the productivity gradient to the structure of the species pool.

Parameter	Modality (if categorical)	Position of diversity peak		Average diversity		Coefficient of variation of diversity	
		Estimate	Partial η^2^	Estimate	Partial η^2^	Estimate	Partial η^2^
Intercept	/	0.494	/	8.44	/	0.603	/
Distribution of *f*_*i*_	Decreasing	**−0.291**	**0.617**	0.369	0.080	**0.00841**	**0.667**
	Bell-shaped	**−0.116**		−0.685		**0.2426**	
	Increasing	**0.0910**		−1.39		**0.268**	
*N*		3.48 × 10^−5^	<0.001	**0.0759**	**0.672**	4.38 × 10^−4^	0.043
Mean of *f*_*i*_		**0.326**	**0.411**	−0.454	0.00328	**0.204**	**0.305**
Range width of *f*_*i*_		**−0.193**	**0.137**	0.182	0.0330	**−0.609**	**0.710**
Mean of *l*_*i*_		0.0378	0.0761	−0.731	0.00690	0.00260	0.001
Range width of *l*_*i*_		**−0.408**	**0.647**	−2.15	0.110	−0.00320	<0.001
*Multiple R* ^*2*^		*0.815*		*0.700*		*0.832*	

Estimates and partial η^2^ statistics associated with each parameter are displayed. Predictors with partial η^2^ > 0.15 are shown in bold and discussed in the main text.

### Replicating BEF experiment settings

Biodiversity–ecosystem functioning experiments have systematically found a positive relationship between biodiversity and productivity. These experiments traditionally manipulate the size of the species pool (*N*) by sowing a restricted and increasing number of species^9^. Replicating this approach, using each 3000 simulated species pools, we randomly selected a reduced number of species (1, 5, 10, 20, 50, 100 or 150 ten times each, up to the number of species in the pool) and estimated the diversity of the resulting community after assembly in five resource conditions.

We found a consistent positive relationship between species number and productivity irrespective of resource level because of the stronger competition between conspecifics than between heterospecifics (Fig. 3 – blue lines). The relationship between species diversity and total biomass was always positive (average Spearman correlation: 0.91, range from 0.22–0.998). A larger number of species *N* at the start of the simulation implies the co-existence of higher diversity *n* in the community, which increases the productivity until saturation indicating that over yielding no longer takes place. The discrepancy between the results of BEF experiments and natural systems^37,38^ has fuelled extensive debate^39,40^. The crucial difference between BEF settings and natural communities is that the species number of BEF experimental communities is maintained artificially low compared to the available species pool. In our model, this means that supplying more species increases productivity because there is potential for over-yielding. The level of resources acts in interaction with the functional trait distribution within the species pool to determine the saturation point of productivity. We show how resource limitation and competitive mechanisms can be integrated into a single plausible plant-resource community model that reconcile the positive biodiversity-productivity relationship of BEF experiments^7,41^ with the varying forms of biodiversity-productivity relationships observed in nature^38^ and further formalize concepts earlier illustrated by Schmid^39^.

### Bias in observed species pools in Europe

**Figure 5:**
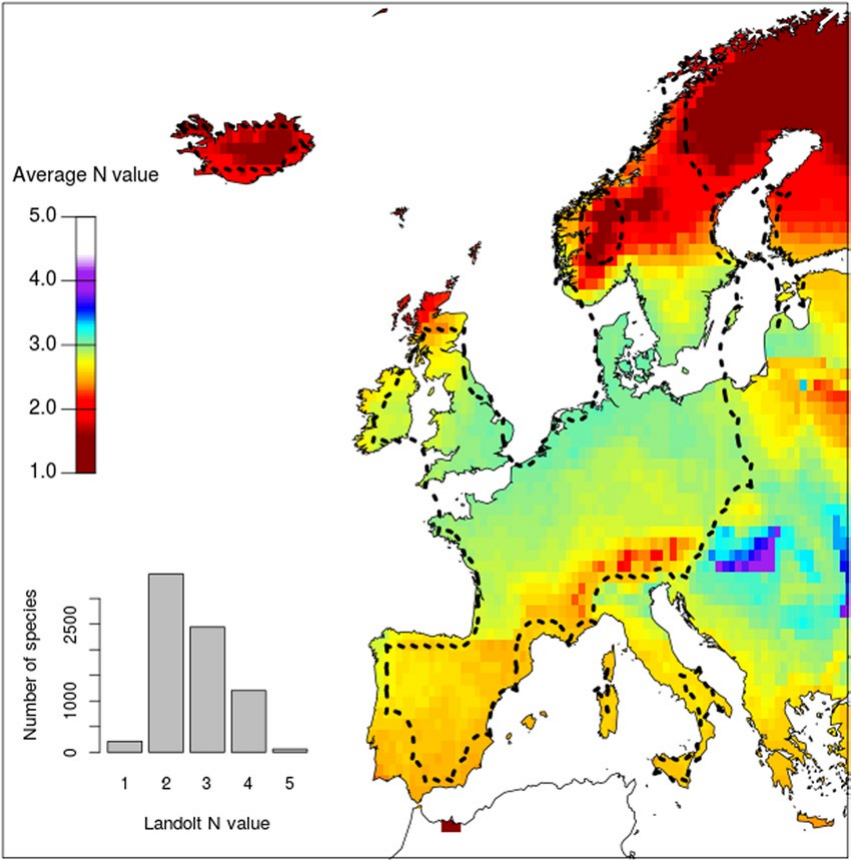
Mean Landolt N nutrient values of the flora across Europe based on the distribution of 6046 species. A low nutrient value indicates that a species is oligotrophic, while a high value indicates a preference for richer soils. The black line delimits the zone in which we estimated that over 50% of the flora was included (see methods and Figure S2) and was calculated with an alpha-hull (α = 1.5). The histogram shows the distribution Landolt N values for 7394 species within the overall European species pool. The map was generated using R3.4.2 (https://cran.r-project.org)^68^. The coastline data was downloaded from the US National Centers for Environmental Information^69^.

So why do we observe a predominant pattern of loss of species diversity with fertilization in nature (rather than bell-shaped or positive^5^), even in low-nutrient environments^42^ where our model would predict an increase in species diversity? Our model predicts that the effect of fertilization should rather lead to variable outcomes depending on the structure of the local species pool; thus an explanation is that local species pools are systematically biased towards species adapted to low level of nutrients and intolerant to competition. To test that hypothesis, we assembled a database of plant species distributions and nitrogen preference indicator values (based on Landolt indicator soil nutrient “N” values^43^). We show that the European species pool is generally biased toward species adapted to oligotrophic environments: over a total of 7394 European species, 49.9% had an indicator value of 1 or 2 among the five categories, which corresponds to a preference for “oligotrophic” to “very oligotrophic” soils (Fig. 5). Hence, the frequency distribution of this ecological trait is biased towards species preferring low-nutrient soils. Moreover, across Europe, based on the mapping of 6046 species distributions, 79% of the 5° cells had a flora with an average Landolt resource indicator value inferior to 3 showing that local species pools are also biased towards low nutrient soil niche preference. This was especially the case for local species pools from mountainous, Mediterranean or high latitude environments, which had on average more species adapted to oligotrophic environments compared to the species pool from the European lowlands (Fig. 5). According to our mathematical model, such bias in the species pool toward preferences for poorer soils (low *f*_*i*_) renders assemblages more sensitive to biodiversity loss under fertilization.

## Discussion

We show how, along a resource gradient, the shape of the relationship between diversity and productivity varies according to the trait distribution within the species pool. Our results shed light on the debate about the shape of the diversity–productivity relationship: modulating the size and trait distribution of the species pool influences the shape of the relationship, from uniform, bell-shaped, increasing or decreasing (Fig. 3). Empirical observations of diversity–productivity relationships come from very different environments^24,44^, which implies different species pool structure. Hence, inconsistent results among studies are not surprising in the light of our model: the structure of the species pool is expected to vary greatly from one region to another, and sampling different regions should thus influence the form of the diversity–productivity relationship. Even at the scale of Europe, the structure of species pools strongly varies and according to our model, these geographic differences could foster different local diversity–productivity relationships and blur the signal in geographical studies^24–26^.

Despite geographic variation, we found a general bias in soil resource level preferences in plant species across Europe. Species pool structure at the European scale is likely to be associated with the different regional ecological and evolutionary histories^45–47^, and has consequences for the species response to systematic fertilization of grasslands. An explanation for this bias of species pool is that the commonness of fertile habitats are fairly recent and thus, only a lower number of species are adapted to it^48^. Assuming that the “N” indicator value reflects our trade-off hypotheses^49,50^, our results suggest that the pervasive observations of a decrease in species richness with fertilization^4,5,10^ is explained by natural species pools being generally biased toward oligotrophic species (Fig. 5) as suggested by early theoretical frameworks^28^. Specific regions, especially mountain grasslands, might be particularly susceptible to species loss with increasing resource availability, a claim supported by empirical observations^42^. Resource input in such systems is expected to lead to the predominance of the few copiotrophic species in the pool and to a general decline of the diversity in grasslands.

The model allows the reconciliation of positive relationships in classical BEF experiments^23^ with observations from natural systems varying from positive to negative^37–39^. Consistent with experiments^8^, the architecture of our model reproduced the positive relationship between diversity and productivity due to over-yielding. At constant resource levels when the size of species pool is manipulated, the inclusion of more species promotes greater productivity until saturation. In contrast, along a resource gradient, the diversity depends on both resource levels and the species pool structure, creating a hump-shaped relationship. Overall, our results are contingent on the existence of trade-offs among the main plant functions: growth *f*_*i*_, mortality *m*_*i*_, intra-specific *c*_*i*_ and inter-specific competition *l*_*i*_. The trade-off between growth and mortality is supported by overwhelming evidence^18,32^, but the existence of the other trade-offs (with competition tolerance) should be further documented.

Including the main ecological processes involved in the relationship between resources, diversity and productivity^11,15,51^, we provide a synthesis of the mechanisms shaping diversity–productivity relationships in grasslands. Our study calls for a better understanding – and experimental treatment – of the interplay between the potential species pool of a focal community and the structure of the realized community. Our results also warn against taking knowledge garnered from experiments and applying this directly for the management of natural systems, since experimental conditions, e.g., the number of species, might greatly differ between natural and manipulated systems. Future investigations should shift toward the link between the structure of species pools and productivity, rather than focusing on species diversity *per se*. Finally, it has been recently advocated that more mechanistic models should be used in biodiversity modeling^52^. We show here how simple theoretical models with relatively few parameters can yield a better understanding of the complex patterns of biodiversity observed in nature in a mechanistic way. Such models provide a first step toward the use of a more mechanistic approach in biodiversity modeling to provide better forecasts under global changes.

## Methods

### Link with the R* of Tilman

From Equation 1, we can deduce the minimum amount of resource that plant species *i* requires in the absence of competition. It can be expressed as the value *R*_*i*_ * at which the relative growth rate of species *i* become positive when the biomass of all species is close to 0:

3 $$\frac{1}{{P}_{i}}{\frac{d{P}_{i}}{dt}}_{\forall j,{P}_{j}=0}={f}_{i}R-{m}_{i}$$

4 $${\rm{Then}},\frac{1}{{P}_{i}}{\frac{d{P}_{i}}{dt}}_{\forall j,{P}_{j}=0}\ge 0\leftrightarrow R\ge {R}_{i}^{\ast }=\frac{{m}_{i}}{{f}_{i}}$$

*R*_*i*_ * represents the tolerance of species *i* to resource depletion. As such, this parameter characterizes a feature of the species abiotic niche: for a species to grow in the community in the absence of competition, *R*_*i*_ * should be below the resource capacity *S*.

### Analysis of the effect of trade-offs

Each species is characterized by four theoretical traits: *f*_*i*_ *, m*_*i*_ *, l*_*i*_ and c_i_. We defined five possible sets of trade-offs that could constrain species trait variability.

**1)No trade-off:** species traits are determined by independent uniform distributions that depend only on the range of each trait.

**2)Mortality–Resource absorption trade-off:** species are placed on an axis that contrasts fast-growing (high *f*_*i*_), stress-sensitive (high *m*_*i*_) species with high *R*_*i*_ * to slow-growing (low *f*_*i*_), stress-tolerant (low *m*_*i*_) species with low R_i_*.

**3)Mortality–Biomass tolerance trade-off:** species are placed on an axis that contrasts stress-sensitive (high *m*_*i*_), and biomass-tolerant (low *l*_*i*_) species to stress-tolerant (low *m*_*i*_) but biomass-intolerant (high *l*_*i*_) species.

**4)Resource absorption–Biomass tolerance trade-off:** species are placed on an axis that contrasts fast-growing (high *f*_*i*_) and biomass-tolerant (low *l*_*i*_) species to stress-tolerant (low *m*_*i*_) but biomass-intolerant (high *l*_*i*_) species.

**5)Mortality–Resource absorption–Biomass tolerance trade-off:** species are placed on an axis that contrasts fast-growing (high *f*_*i*_), stress-sensitive (high *m*_*i*_), competition tolerant species that grow only in resource-rich environments (high R_i_*), and slow-growing (low f_i_), stress-tolerant (low m_i_), competition intolerant (high l_i_) species that can grow in resource-poor environments (low R_i_*). This represents a single functional axis (all traits are perfectly correlated) and is analog to the “competition”–“stress-tolerant” side of the Grime triangle^18^. In addition, to the trade-offs defined by Equation 3, we defined l_i_ as being linearly related to R_i_* and f_i_.

Details about the mathematical implementation of the trade-offs are available in the supplementary methods.

We evaluated the role of the trade-offs on species co-existence and turnover along the resource gradient by generating 500 species pools for each trade-off structure. Other species pool parameters (see Table S1) were kept fixed at the average of their distribution, and the distribution of species trait along trade-off axis was uniform.

### Analysis of the effect of species pool structure

We performed this analysis only in the situation of a trade-off between mortality, resource absorption and biomass tolerance.

### Parameters of variation

To analyse the influence of the species pool structure on the diversity–productivity relationship, we defined distributions for the following parameters structuring the species pool:

- Number *N* of species in the species pool

- Distribution of *f*_*i*_: we tested the impact of four shapes of the distribution of f_i_ values within a specified range (see below): a uniform distribution, or a triangular distribution with the mode of the distribution being either the minimum (decreasing distribution), center (bell-shaped distribution) or maximum (increasing distribution) of the range.

- Average of parameter *f*_*i*_

- Range width of parameter *f*_*i*_

- Average of parameter *l*_*i*_

- Range width of parameter *l*_*i*_

### Analysis of community structure along a resource gradient

To analyse the influence of the soil resource parameters on community assembly, we first drew the species pool parameters from uniform distributions (Table S1 for their respective ranges). We then drew at random species traits under those constraints, from either a uniform, decreasing, bell-shaped or increasing distribution. We studied community assembly for different values of the soil resource parameters *a* and *S* (Table S1). In total, we tested the assembly of 3000 species pool structures under 42 different soil resource conditions.

We solved the differential equation system using the ordinary differential equation solver ODE45 (Matlab). We then characterized the relationship between community diversity calculated with the logarithm of the inverse of Simpson^53^ and community total biomass $(\sum _{i}{P}_{i})$ using a generalized additive model. We described the modelled curve using the following three statistics:

(1) the average diversity across the productivity gradient.

(2) the coefficient of variation of diversity across the productivity gradient.

(3) β-diversity across the productivity gradient. It was calculated as the ratio of the diversity of the averaged relative abundance of species across communities (γ-diversity) and the averaged diversity of communities (α-diversity). The metric was chosen because of its independent on the α-diversity^53,54^.

(4) the relative position of the maximum of the modelled relationship along the productivity gradient. If the relationship was decreasing, then it was close to 0, if the relationship was increasing, then it was close to 1 and if the relationship was bell-shaped, then it took its value between 0 and 1.

We then related these three statistics to species pool parameters using a linear model.

### Replicating BEF experiment settings

In each of the previously defined species pools, we selected four resource levels: (a,S) = {(0.05,0.1); (0.85,1.1); (1.65,2.1); (2.45,3.1)}. For each resource level, we randomly selected a set of 1, 5, 10, 20, 50, 100 or 150 species (up to the size of the species pool), ten times each. We solved the differential equation system using the ordinary differential equation solver ODE45 (Matlab) and calculated the diversity and total biomass of the simulated community, then computed the Spearman correlation between diversity and biomass to test if the relationship was increasing or decreasing.

### Species pool structure across Europe

To describe the grassland species pool structure across Europe, we combined four databases of expert-based ecological indicator values for European plant species: the Swiss flora database provided by Flora Indicativa^43^, the Ellenberg database for the Central European flora^55^, the Italian flora niche indicators^56^ and the British Isles flora niche indicators^57^. More details are available in the supplementary materials. We used the indicator “N” that characterises the species ecological preference for soil resource levels^58,59^ and ranges from 1 (very oligotrophic, e.g., *Viola rupestris*) to 5 (very rich to over-fertilized, e.g., *Urtica dioica*). For all species with indicator values, we mapped their distribution across Europe using information from the Global Biodiversity Information Facility^60^. Synonymies were handled with the R-package *taxize* ^61^ based on the nomenclature of GBIF^62^ and all information was extracted and aggregated at the species level. GBIF data^60^ was extracted using the R-package *rgbif* ^[ 63^. We constructed distribution maps for each species. We cleaned species occurrences by deleting data from outside of the area of interest and excluding isolated points and species with too few occurrences (fewer than 20). We divided Europe into eleven parts corresponding to the main biogeographical zones (source: European Environment agency, Figure S1) to integrate possible discontinuous species distributions (for instance due to mountain ranges). In each biogeographic zone, we defined a convex polygon surrounding species occurrences. Then we refined each species distribution map by removing areas where temperature fell outside the minimum or maximum temperature range of the species. To estimate the species temperature range we defined a climatic envelop by crossing species occurrences with a mean temperature map estimated from twelve maps of current conditions downloaded from Worldclim^64^. Final distribution maps of well-known species were reviewed visually. We obtained an atlas containing the distributions of 6133 species that we aggregated on a 0.5° resolution grid covering Europe.

Because of the sampling bias present within the GBIF database^65^ and in our combined database of species niche indicators, we mapped the area of Europe on which our approach is more accurate. We compared the species pool across Europe predicted by our approach to the prediction given by the Atlas Florae Europaeae^66^ (AFE, vol. 1**–**13). The AFE provides the presence and absence of plant species in a 50 by 50 km grid across Europe but is restricted to some plant families (including Caryophyllaceae, Brassicaceae) but missing important families in grasslands (e.g. Poaceae). At each grid point, we extracted the presence or absence of the AFE species list based on our distribution maps. We then recorded the percentage of the local flora based on the AFE that was present according to our approach. Overall, our analysis suggested that the flora of the Northern European plain was well sampled by our approach (over 80%, Figure S2) and decreased gradually in quality in more peripheral areas (particularly Eastern Europe), as well as particular environments (coasts and some Mediterranean climate areas, Fig. 5) where less than 50% of the flora was well sampled. To quantify the bias in Landolt N values in species pools across Europe, we calculated the average Landolt value at each location. We simply used a mean rather than more specific indicators of distribution bias such as the mode of the distribution or its skewness because these metrics were heavily correlated to the mean (r = 0.72 and −0.83, respectively, after discarding areas with less than 20 modelled species).

Simulations were computed using MATLAB^67^. Figures and maps were generated using R3.4.2^68^.

### Data avilability

Data for this article has been available at 10.6084/m9.figshare.5573560.v1.

## Electronic supplementary material

Supplementary materials
