# Supplementary material for: Species pool distributions along functional trade-offs shape plant productivity–diversity relationships
Source: Sci Rep. 2017 Nov 13;7:15405. doi: 10.1038/s41598-017-15334-4 (PMC5684142; doi:10.1038/s41598-017-15334-4)

Species pool distributions along functional trade-offs shape plant productivity–diversity relationships: supplementary materials

L. Chalmandrier, C. Albouy and L. Pellissier

Supplementary methods

I Possible trait trade-offs in the theoretical model

Each species is characterized by four theoretical traits: f_i, m_i, l_i et c_i. We defined five possible trade-offs situations that can constrain species trait variability.

a) No trade-off: species traits are determined by independent uniform distributions that depend only on the range of each trait. We nonetheless establish the following constrain to keep species R^*_i between 0 and 1.

$$m_i < f_i \qquad \text{(Equation S1)}$$

b) Mortality – Resource absorption trade-off: species are placed on an axis that contrast fast-growing (high f_i), stress-sensitive (high m_i) to slow-growing species (low f_i), stress-tolerant (low m_i). We defined a power law relationship between f_i and m_i of exponent B = 0.5 so that fast-growing, stress-sensitive species have a high R_i^* and are not able grow in resource-poor environments.

$$f_i = A \times m^{1/2} \iff R_i^* = A^{-2} \times f_i \iff R_i^* = A^{-1} \times m^{1/2} \qquad \text{(Equation S2)}$$

c) Mortality - Resource absorption – Biomass tolerance trade-off: species are placed on a axis that contrasts fast-growing (high f_i), stress-sensitive (high m_i) and that grow only in resource-rich environment (high R_i^*) and slow-growing species (low f_i), stress-tolerant (low m_i), competition intolerant species (high l_i) that can grow in resource-poor environment (low R_i^*). This represents a single functional axis (all traits are perfectly correlated) and is analog to the “competition”- “stress-tolerant” side of the Grime triangle (Grime 1977). In addition, to the trade-off defined by Equation 3, we defined l_i as being linearly related to R_i^* and f_i

$$l_i = C \times R_i^* + D \qquad \text{(Equation S3)}$$

And thus:

$$l_i = \frac{C}{A} \times m^{1/2} \qquad \text{(Equation S4)}$$

$$l_i = \frac{C}{A^2} \times f_i + D \qquad \text{(Equation S5)}$$

4) Mortality – Biomass tolerance trade-off: species are placed on an axis that contrast stress-sensitive (high m_i) and biomass-tolerant species (low l_i) to stress-tolerant (low m_i) but biomass-intolerant species (high l_i). This trade-off was defined using only Equation 1 and Equation 5.

5) Resource absorption – Biomass tolerance trade-off: species are placed on an axis that contrast fast-growing (high f_i) and biomass-tolerant species (low l_i) to stress-tolerant (low m_i) but biomass-intolerant species (high l_i).This trade-off was defined using only Equation 1 and Equation 6.

Relationships among traits are defined by the parameters A, C, D, F et E. They control the range of numerical values that traits take relative to each other. A, C, E are strictly positive and D and F are positive. We calibrated these parameters values according to the range that could take f_i, m_i and R_i* (see Table S1)

II Database of species nutrient niche indicators for maps of European Flora

We combined four databases of niche indicators for European plant species: the Swiss flora database provided by Flora Indicativa[1], the Ellenberg database for Central Europe flora[2], the Italian flora niche indicators[3] and the British Islands flora niche indicators[4]. The indicator "N" characterized species niche position along local productivity gradient[5,6]. Possible overlap between such databases have been discussed in the literature[5,7] and establish that there is limits to the extent on which one can use the indicator scale of one geographic area into another. However, in the absence of an extensive community database to calibrate the relationship between databases, we used simple linear relationships to estimate the niche indicator value of species along the Swiss Landolt niche indicator scale from Flora Indicativa. This scale was preferred to the others because it contains less classes (five against nine for the other databases); values were available for a large number of species (6133) typical of areas well sampled in Europe by GBIF[8] (compared to Italy for instance, Figure S2).

We constructed a set of linear models predicting the Landolt value from every possible combinations of other available niche indicators. Overall, the models exhibited a good coefficient of determination (around 0.60) or moderate for the model for species with only an indicator value in the Italia flora database (0.38). We then used these models to predict the missing Landolt values for each species

according to their available niche indicators values (Table S2). Finally species predicted indicator values were rounded to the closest integer.

References

1. Landolt, E. & Bäumler, B. *Flora indicativa: Ökologische Zeigerwerte und biologische Kennzeichen zur Flora der Schweiz und der Alpen*. (Haupt, 2010).
2. Ellenberg, H. *et al.* Zeigerwerte von pflanzen in Mitteleuropa. (1992).
3. Pignatti, S., Menegoni, P. & Pietrosanti, S. Biondicazione attraverso le piante vascolari. Valori di indicazione secondo Ellenberg (Zeigerwerte) per le specie della Flora d'Italia. *Braun-Blanquetia* **39,** 97 (2005).
4. Fitter, A. & Peat, H. The Ecological Flora Database. *J. Ecol.* **82,** 415–425 (1994).
5. Hill, M. O., Roy, D. B., Mountford, J. O. & Bunce, R. G. H. Extending Ellenberg's indicator values to a new area: an algorithmic approach. *J. Appl. Ecol.* **37,** 3–15 (2000).
6. Schaffers, A. P. & Sýkora, K. V. Reliability of Ellenberg indicator values for moisture, nitrogen and soil reaction: a comparison with field measurements. *J. Veg. Sci.* **11,** 225–244 (2000).
7. Godefroid, S. & Dana, E. D. Can Ellenberg's indicator values for Mediterranean plants be used outside their region of definition? *J. Biogeogr.* **34,** 62–68 (2007).
8. GBIF.org (29th February 2016) GBIF Occurrence Download http://doi.org/10.15468/dl.ywhpmz
9. R Core Team. *R: A Language and Environment for Statistical Computing*. (R Foundation for Statistical Computing, 2017).
10. Wessel, P. & Smith, W. H. F. *Global Self-consistent Hierarchical High-resolution Geography Database*. (2013).

Table S1. Model parameter values for the study of the impact of species pool structure on the productivity-diversity relationship.

Name	Abbreviation	Feature	Studied range
**Species pool structure parameters**			
Number of species	N		[50, 200]
Resource uptake rate	f_i	Average	[1.1, 2.1]
		Range width	[0.4, 2.0]
Effect of neighbouring biomass	l_i	Average	[1.6, 2.5]
		Range width	[0.4, 3.0]
Mortality rate	m_i		Deduced from the other parameters
Intraspecific competition rate	c_i	Average	0.6
		Range width	0
Shape of the trade-off between mortality and resource absorption	B		0.5
Tilman's R*	R_i*		[0, 1]
**Soil resource parameters**			
Resource renewing rate	a		{0.05, 0.55, 1.05,…, 2.55}
Maximum resource capacity	S		{0.1, 0.6, 1.1,…, 3.1}

Table S2. Characteristics of the linear models used to bring all Landolt indicator values on the same scale. The Ellenberg (CE), Pignatti (IT) and Fitter (UK) values records preferences for soil resources on a scale from 1 to 10, while Landolt value (CH) on a scale from 1 to 5. We used species with several classifications to bring N values on the same 1 to 5 scale.

Available traits	Number of species used to fit the model (species in common with the Landolt database)	R^2 of the linear model	Number of predicted species indicator values
IT, UK, CE	1057	0.66	67
IT, CE	2005	0.58	73
EL, UK	1133	0.65	18
IT, UK	1255	0.63	153
IT	3659	0.38	1950
UK	1415	0.61	135
CE	2175	0.57	41

Figure S1. Map of the main biogeographical zones of Europe used to modelled plant species distributions (source: European Environment agency, http://www.eea.europa.eu/data-and-maps/figures/biogeographical-regions-in-europe-1). The map was generated using R3.4.2 (https://cran.r-project.org)[9].

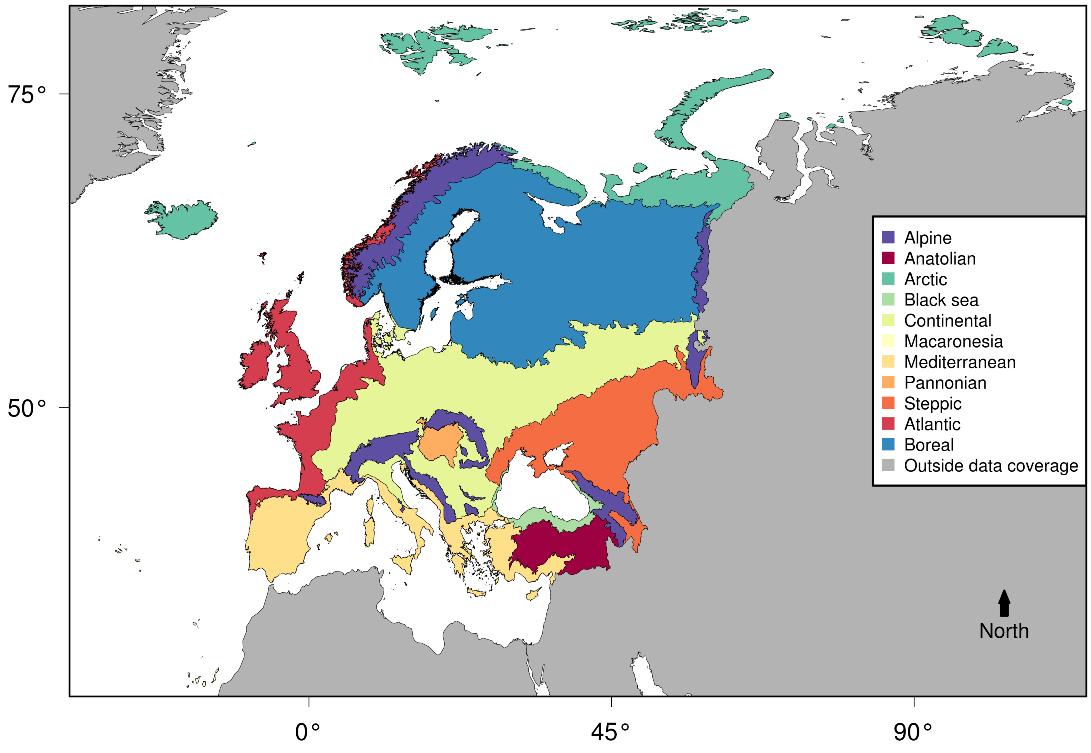

Figure S2. Percent of species across the European species pools modelled with our methodology that match the Atlas Flora Europeae for each 1° cell across Europe. The comparison was done for the families listed in volume 1-13 of Atlas Flora Europeae (https://www.luomus.fi/en/list-families-mapped-atlas-florae-europaeae). The map was generated using R3.4.2 (https://cran.r-project.org)[9]. The coastline data was downloaded from the US National Centers for Environmental Information10.

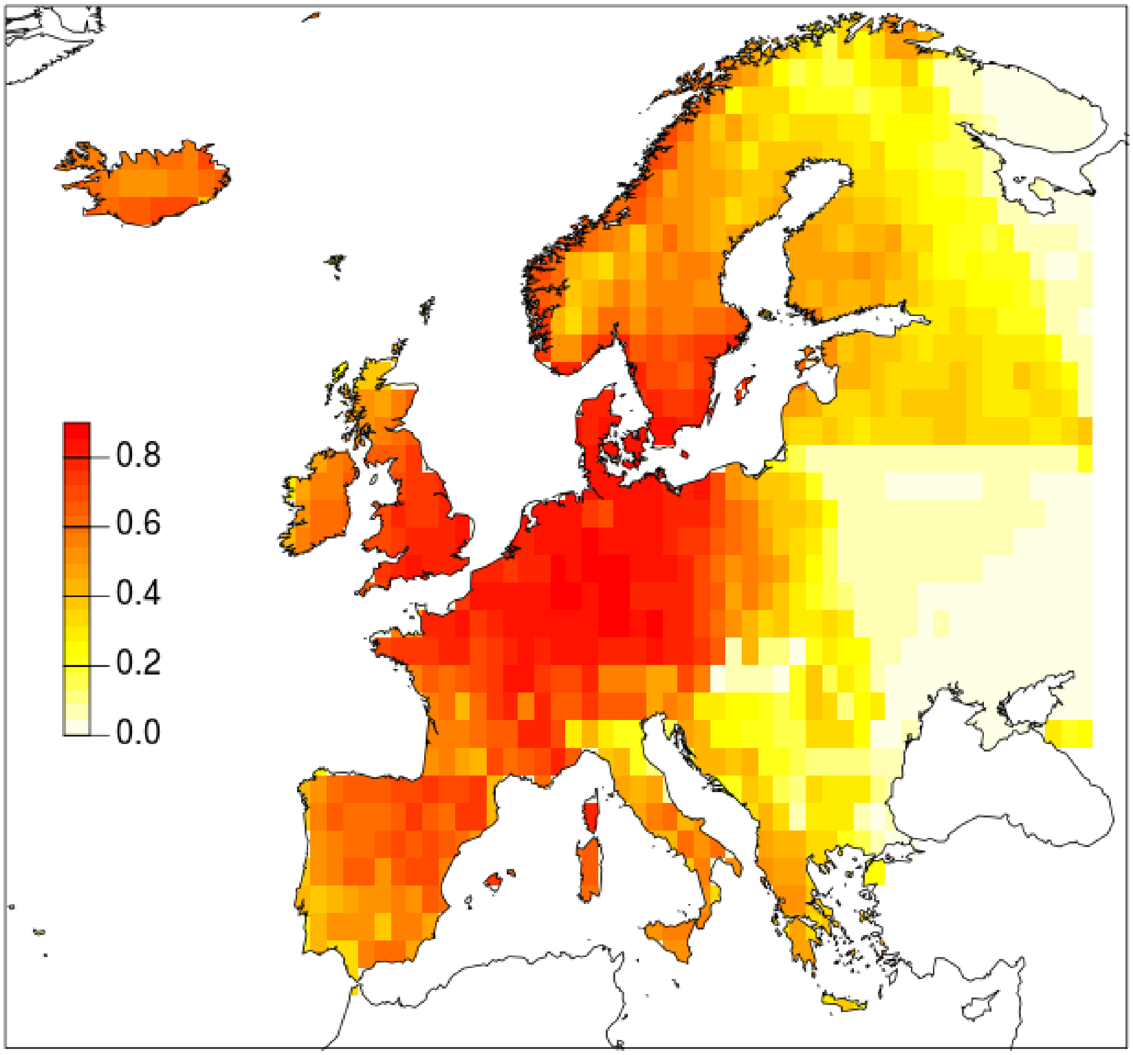

Supplement: Supplementary file 1 — Supplementary materials [file 41598_2017_15334_MOESM1_ESM.pdf]
